# Self-assemblies of γ-CDs with pentablock copolymers PMA-PPO-PEO-PPO-PMA and endcapping via atom transfer radical polymerization of 2-methacryloyloxyethyl phosphorylcholine

**DOI:** 10.3762/bjoc.11.247

**Published:** 2015-11-23

**Authors:** Jing Lin, Tao Kong, Lin Ye, Ai-ying Zhang, Zeng-guo Feng

**Affiliations:** 1 School of Materials Science and Engineering, Beijing Institute of Technology, Beijing 100081, China

**Keywords:** ATRP, γ-CD, double-chain-stranded, pentablock copolymer, poly(pseudorotaxane), polyrotaxane, single-chain-stranded

## Abstract

Pentablock copolymers PMA-PPO-PEO-PPO-PMA synthesized via atom transfer radical polymerization (ATRP) were self-assembled with varying amounts of γ-CDs to prepare poly(pseudorotaxanes) (PPRs). When the concentration of γ-CDs was lower, the central PEO segment served as a shell of the micelles and was preferentially bent to pass through the γ-CD cavity to construct double-chain-stranded tight-fit PPRs characterized by a channel-like crystal structure. With an increase in the amount of γ-CDs added, they began to accommodate the poly(methyl acrylate) (PMA) segments dissociated from the core of the micelles. When more γ-CDs were threaded and slipped over the segments, the γ-CDs were randomly distributed along the pentablock copolymer chain to generate single-chain-stranded loose-fit PPRs and showed no characteristic channel-like crystal structure. All the self-assembly processes of the pentablock copolymers resulted in the formation of hydrogels. After endcapping via in situ ATRP of 2-methacryloyloxyethyl phosphorylcholine (MPC), these single-chain-stranded loose-fit PPRs were transformed into conformational identical polyrotaxanes (PRs). The structures of the PPRs and PRs were characterized by means of ^1^H NMR, GPC, ^13^C CP/MAS NMR, 2D ^1^H NOESY NMR, FTIR, WXRD, TGA and DSC analyses.

## Introduction

Cyclodextrins (CDs) are a series of macrocyclic molecules composed of 6, 7, or 8 (α-, β-, and γ-CD, respectively) glucopyranose units. Their hydrophilic surface and hydrophobic inner cavity character and deformable cavity size allow for the self-assembly or inclusion of various polymer chains to generate poly(pseudorotaxanes) (PPRs) or polyrotaxanes (PRs) after endcapping with bulky stoppers. Since Harada et al. first reported the α-CD-based single-chain-stranded PPRs constructed from the inclusion complexation of α-CDs with poly(ethylene glycol) (PEG) [1], a great variety of polymers with different cross-sectional areas have been shown to thread CDs to create PPRs. For example, β-CDs are single-chain-stranded with poly(propylene glycol) (PPG) but not with PEG [2–3], and γ-CDs are not only single-chain-stranded with poly(methyl vinyl ether) (PMVE) [4] or poly(dimethylsiloxane) (PDMS) [5], but also double-chain-stranded with PEG and poly(ε-caprolactone) (PCL) [6]. Recently, Akashi et al. reported the single-chain-stranded inclusion complexation of γ-CDs with poly(methyl methacrylate) (PMMA) [7] and poly(methacrylic acid) (PMAA) [8]. It is worth noting that there is a significant correlation between the size of the CD cavity and the cross-sectional area of the fitting polymers. Accordingly, all the aforementioned PPRs and PRs were created from a matched recognition between the CD cavities and incoming polymer chains, showing the typical channel-like tight-fit crystal structure [1–8]. Due to the fantastic, mechanically interlocked architecture, these PPRs and PRs can be employed as candidates or precursors for complex supramolecular assemblies to realize novel functions [9].

In comparison to α- and β-CDs, γ-CDs possess a larger inner cavity diameter and a higher structurally deformable and adaptable capacity [10]. Recently, studies towards the synthesis and characterization of novel γ-CD-based unmatched PPRs and PRs have attracted attention for their unusual loose-fit and/or over-fit inclusion complexation structure other than the channel-like tight-fit structure [10–12]. It was shown that when self-assembling with PHEMA-PPO-PEO-PPO-PHEMA, γ-CDs could be threaded onto and moved over the 2-hydroxyethyl methacrylate (PHEMA) segments to form a mixed single-chain-stranded loose-fit (PEO) and over-fit (PHEMA) architecture [13]. Using in situ ATRP, those PPRs were successfully transformed into the same conformational PRs [14]. Owing to a more flexible or retardant movement of the entrapped γ-CDs along the thinner PEO and thicker PHEMA segments (as compared with PPO or PMAA), respectively, these unmatched PRs possess the potential to be applied as dynamic-responsive materials, carriers for controlled drug release, biosensors and catalysts.

**Scheme 1:**
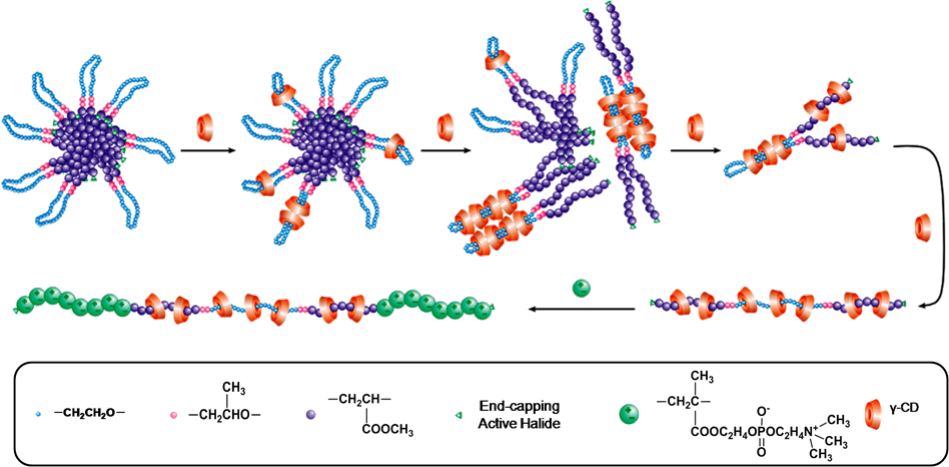
Schematic description of self-assembly of γ-CDs with PMA-PPO-PEO-PPO-PMA and one-pot endcapping via in situ ATRP of MPC.

Poly(methyl acrylate) (PMA) prepared via ATRP of methyl acrylate (MA) is a typical hydrophobic polymer with a more flexible main chain and smaller cross-sectional area as compared with PHEMA. Attaching PMA to two ends of PPO-PEO-PPO imparts the resulting amphiphilic copolymers with a unique core–shell micellar structure, showing different self-assembly behavior as compared with that of PHEMA-PPO-PEO-PPO-PHEMA. Therefore, a series of PMA-PPO-PEO-PPO-PMA pentablock copolymers were first prepared in this study via ATRP of MA using 2-bromoisobutyryl endcapped PPO-PEO-PPO as a macroinitiator, and then allowed to self-assemble with a varying amount of γ-CDs in aqueous solution. To further highlight the supramolecular architecture of the resulting PPRs, the second in situ ATRP of 2-methacryloyloxyethyl phosphorylcholine (MPC) was conducted to endcap them into the same conformational PR-based multiblock copolymers. A schematic description on the self-assembly evolution of varying amounts of γ-CDs with PMA-PPO-PEO-PPO-PMA and the one-pot endcapping via in situ ATRP of MPC is shown in Scheme 1.

## Results and Discussion

### Preparation of PPRs and PRs

**Scheme 2:**
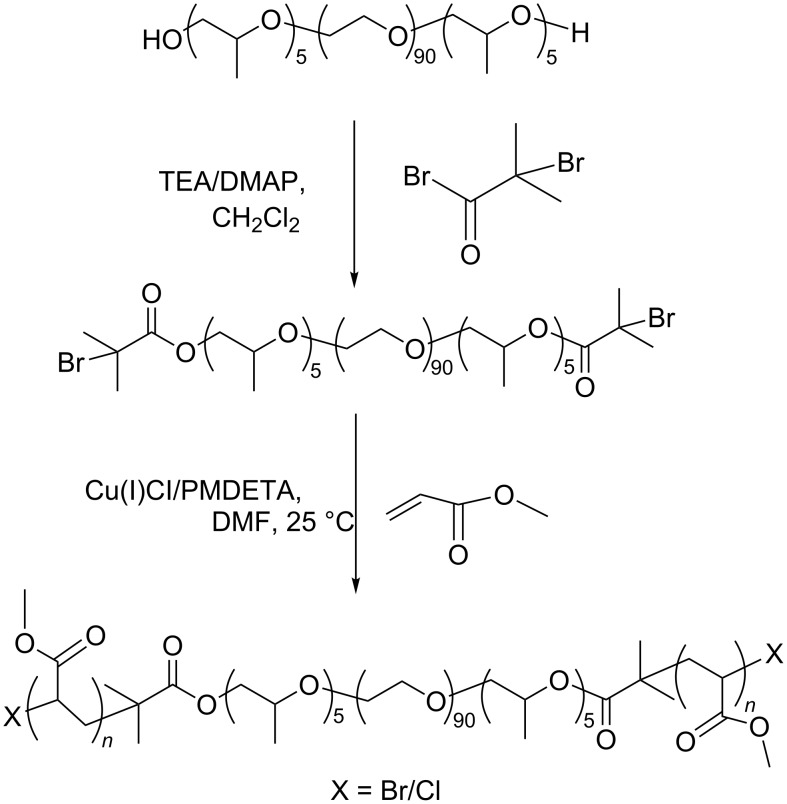
Synthetic pathway of PMA-PPO-PEO-PPO-PMA.

The synthesis of PMA-PPO-PEO-PPO-PMA via ATRP of MA is described in Scheme 2. With the goal of achieving a desired *M*_w_, a narrow PDI and a high preservation of the active Br-terminals for the second in situ ATRP of MPC, the first ATRP of MA was carried out at room temperature using DMF as a solvent and Cu(I)Cl/N,N,N’,N”,N”-pentamethyldiethylenetriamine (PMDETA) as the catalyst.

The macroinitiator and resulting pentablock copolymers are designated as BrPEPBr and PEPxM, respectively, where x is the feed molar ratio of MA to PPO-PEO-PPO. The products were characterized by means of ^1^H NMR and GPC analyses (Figure S1 and Figure S2, Supporting Information File 1). The degree of esterification of BrPEPBr determined by ^1^H NMR was >99%. Additionally, the GPC curves of PEPxM presented a nearly symmetrical monomodal distribution with a narrow polydispersity index (PDI). These results suggested that the target PMA-PPO-PEO-PPO-PMA pentablock copolymers were obtained. The compositions, GPC data and yields are summarized in Table 1.

**Table 1:** Compositions, GPC data and yields of PMA-PPO-PEO-PPO-PMA.

Entry	Molar ratio of BrPEPBr:MA		*M*_n_^a^	*M*_n_^b^	*M*_w_/*M*_n_^b^	Yield/%^c^
	Feed ratio	Found ratio^a^				

BrPEPBr	1:0	1:0	4880	4750	1.15	81.5
PEP40M	1:40	1:22	6770	6880	1.27	65.9
PEP60M	1:60	1:35	7888	8085	1.47	67.5
PEP100M	1:100	1:54	9522	10453	1.31	62.3

^a^Determined by ^1^H NMR analysis in CDCl_3_/DMSO-*d*_6_ (1:1, v/v). ^b^Determined by GPC in DMF using PS standards. ^c^Calculated based on the product weight divided by the raw material weight.

Pentablock copolymers were selected to self-assemble with γ-CDs to prepare PPRs in aqueous solution. The resulting PPRs are designated as PEPxMyCD, where x again is the feed molar ratio of MA to PPO-PEO-PPO and y is the feed molar ratio of γ-CD to PMA-PPO-PEO-PPO-PMA. The macroinitiator was also employed to fabricate the PPR with γ-CDs. Its self-assembled product is designated as PEP15CD, meaning the feed molar ratio is 1:15 for BrPEPBr:γ-CD. The compositions and yields are summarized in Table 2. As can be seen, in addition to good yields in the range of 62.5–73.2%, the resulting molar ratios of PEPxM to γ-CD in the PPRs (including PEP15CD) perfectly matched the feed values, demonstrating the good inclusion complexation ability between γ-CD and pentablock polymers.

**Table 2:** Compositions and yields of PPRs.

Entry	Guest molecule	Molar ratio of guest molecule:γ-CD		Yield/%^b^
		Feed ratio	Found ratio^a^	

PEP40M10CD	PEP40M	1:10	1:11	68.5
PEP40M15CD		1:15	1:15	65.7
PEP40M30CD		1:30	1:23	66.4
PEP60M10CD	PEP60M	1:10	1:11	68.5
PEP60M15CD		1:15	1:16	69.5
PEP60M30CD		1:30	1:23	70.0
PEP100M10CD	PEP100M	1:10	1:12	62.5
PEP100M15CD		1:15	1:15	68.4
PEP100M30CD		1:30	1:24	66.7
PEP15CD	BrPEPBr	1:15	1:17	73.2

^a^Determined by ^1^H NMR analysis in CDCl_3_/DMSO-*d*_6_ (1:1, v/v). ^b^Calculated based on the product weight divided by the raw material weight.

**Figure 1:**
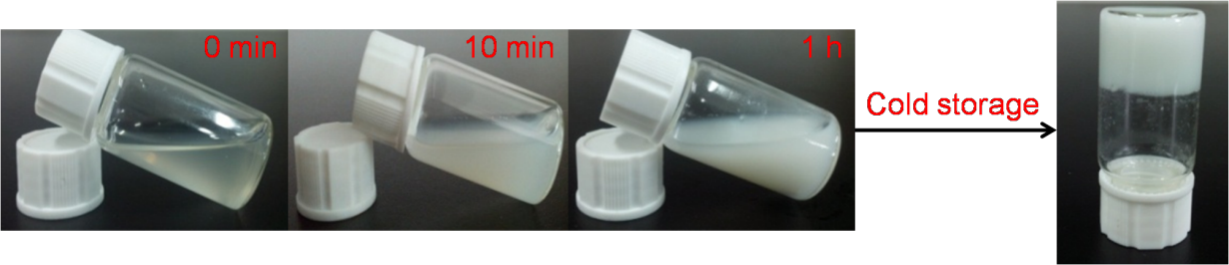
Photographs of the formation of a PEP100M15CD hydrogel.

As a typical example, the self-assembly evolution of PEP100M15CD is shown in Figure 1. When an aqueous solution of saturated γ-CD was mixed with a preset amount of PMA-PPO-PEO-PPO-PMA, the mixture immediately turned turbid, suggesting that the self-assembly of γ-CDs with the incoming polymer chain proceeded similar to the case of inclusion complexation of γ-CDs with the macroinitiator BrPEPBr [13]. Thereafter, the turbidity of the solution quickly increased as more γ-CDs were entrapped on different polymer segments. Surprisingly, a white gel was formed after the turbid mixture was stored at 6–8 °C for at least 24 h. The turbidity evolution and hydrogel formation were observed in other PPR samples, except that PEP15CD was rapidly precipitated from the mixture.

To endcap PPRs into PRs, the PEP40M30CD constructed from the self-assembly of PEP40M with γ-CD at a feed molar ratio of 1:30 was used as a supramolecular initiator. The one-pot ATRP of MPC was conducted in water at room temperature for 60 h using Cu(I)Br/tris(2-(dimethylamino)ethyl)amine (Me_6_TREN) as a catalyst. The resulting PR-based multiblock copolymers are ascribed as PRmCDnP, where m and n stand for the feed molar ratio of γ-CD and MPC to PEP40M, respectively. The compositions, GPC data and yields are summarized in Table 3. As can be seen, the experimental molar ratio of γ-CD to PEP40M varied in the range of 5–8 and a feed molar ratio of 30 was maintained after the second in situ ATRP. However, the degree of polymerization (DP) of PMPC for all the copolymers was increased with the feed molar ratio of MPC to PEP40M, clearly suggesting that PEP40M30CD as an initiator in the form of PPR indeed held a very high degree of chain-end functionality and initiating efficiency for the second ATRP. Relatively lower yields in the range from 27.5–30.5% were evidently caused by the massive slipping of the γ-CDs during the process. Even so, these yields were higher than those any reported loose-fit and/or over-fit γ-CD-based PRs [7–8,10,12].

**Table 3:** Compositions, GPC data and yields of PR-based multiblock copolymers.

Entry	Molar ratio of PEP40M:γ-CD:MPC		*M*_n_ × 10^−4a^	*M*_n_ ×10^−4 b^	*M*_w_/*M*_n_^b^	Yield/%^c^
	Feed ratio	Found ratio^a^				

PR0CD30P	1:0:30	1:0:19	1.23	1.00	1.57	58.9
PR30CD30P	1:30:30	1:5:20	1.91	1.21	1.66	27.5
PR30CD50P	1:30:50	1:6:41	2.66	1.64	1.69	29.4
PR30CD80P	1:30:80	1:8:68	3.72	2.33	1.59	30.5

^a^Determined by ^1^H NMR in DMF-*d*_7_/D_2_O (1:1, v/v) and DMSO-*d*_6_/D_2_O (2:1, v/v). ^b^Determined by GPC in DMF/H_2_O (1:1, v/v) using PEG standards. ^c^Calculated based on the product weight divided by the raw material weight.

### Characterization of PPRs and PRs

#### WXRD measurements

In two recent articles [13–14], we reported that attaching PHEMA to two ends of PPO-PEO-PPO via ATRP of HEMA could change its self-assembly process with γ-CDs. Wherein, the γ-CDs were threaded onto and moved over the PHEMA segments to give access to unmatched γ-CD/PHEMA-PPO-PEO-PPO-PHEMA PPRs showing a mixed loose-fit (with PEO) and over-fit (with PHEMA) architecture, instead of the PEO-bent double-chain-stranded tight-fit ones like those of γ-CD/BrPEPBr PPRs [15–16]. However, attaching hydrophobic PMA to PPO-PEO-PPO renders the resulting amphiphilic copolymers able to form unique polymeric micelles in aqueous solution before the self-assembly with γ-CDs, as compared to PHEMA-PPO-PEO-PPO-PHEMA. The morphology of self-assembled aggregates of PMA-PPO-PEO-PPO-PMA in aqueous solution was observed by TEM. As shown in Figure S3, Supporting Information File 1, the aggregates were formed as spherical or “core–shell” micelles or aggregates of 500–800 nm diameter, where the shell-forming PEO bent segments would effectively bury the PMA core and shield it from water. In general, surface hydrophilic γ-CDs cannot pass through the hydrophilic shell into the core of micelles to include the PMA segments. A schematic description of the self-assembly process of γ-CDs with PMA-PPO-PEO-PPO-PMA is illustrated in Scheme 1.

**Figure 2:**
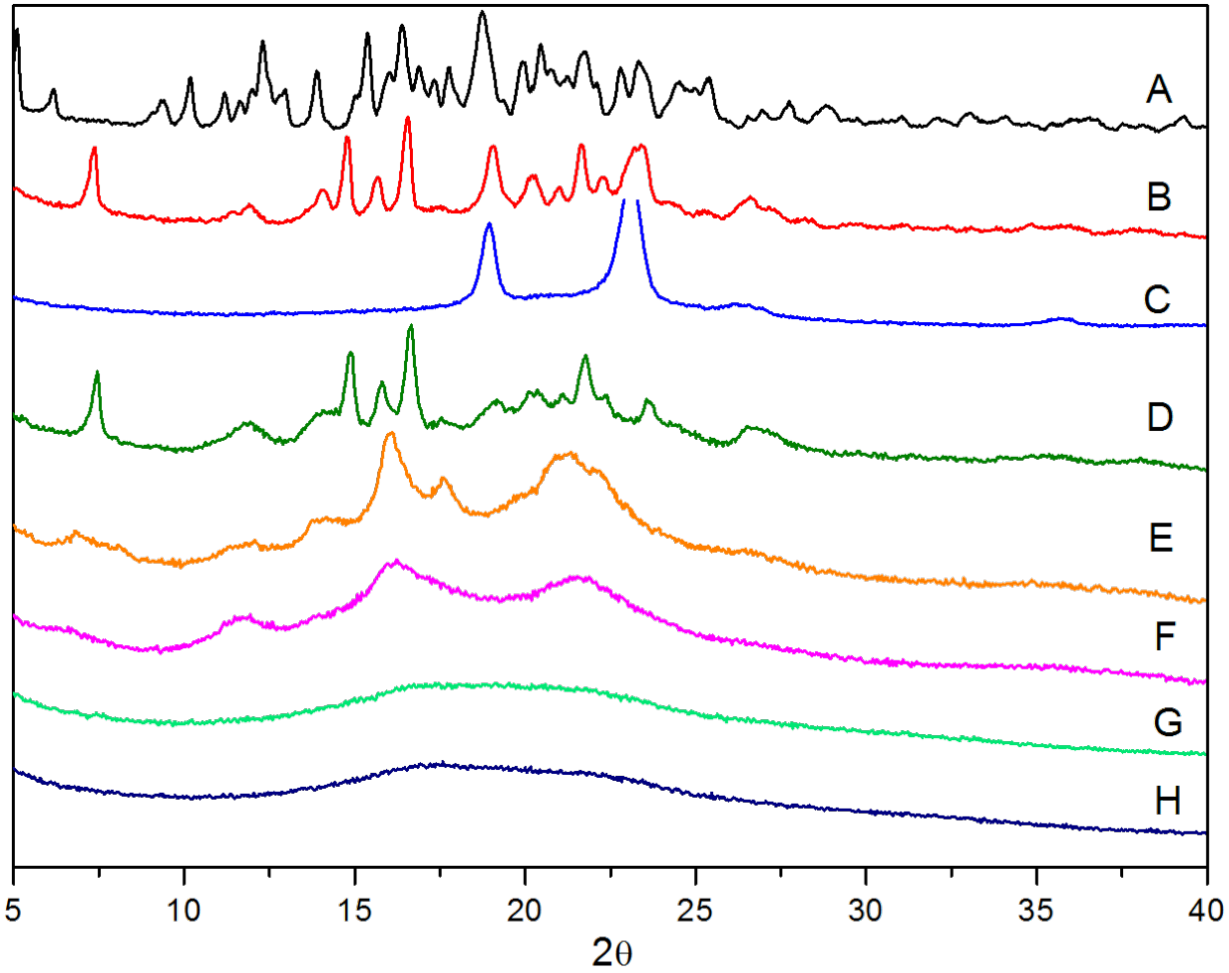
WXRD patterns of γ-CD (A), PEP15CD (B), PEP100M (C), PEP100M10CD (D), PEP100M15CD (E), PEP100M30CD (F), PR30CD50P (G) and PR30CD80P (H).

WXRD measurements provide a powerful tool to analyze the supramolecular structure of the self-assemblies, consisting of varying amounts of γ-CDs with PMA-PPO-PEO-PPO-PMA. As shown in Figure 2, different from the cage-type crystal structure of γ-CD [6], a new diffraction peak at 2θ = 7.5° was clearly observed in the diffraction pattern of PEP15CD self-assembled from the macroinitiator BrPEPBr with γ-CDs. This is characteristic of the PEO-bent double-chain-stranded tight-fit PPRs [17]. In the WXRD patterns of all the PPRs, the disappearance of two prominent peaks at 19.2° and 23.3° of the central PEO segment in the pentablock copolymer PEP100M verified their self-assembly with γ-CDs. Surprisingly, these PPRs presented varying diffraction patterns as a function of γ-CD content. For example, at a feed molar ratio 10:1 of γ-CD to guest pentablock copolymer, PEP100M10CD displayed a pattern similar to PEP15CD as well as to those previously reported [13,15]. This indicates that the central PEO segments were favorably bent to pass through the cavity of γ-CDs to give rise to the double-chain-stranded tight-fit PPR. For example, at a feed molar ratio of γ-CD to PEP100M equal to 30:1, PEP100M30CD depicted three broad peaks at 2θ = 12.4°, 17.2° and 21.5°, instead of a peak at 7.5° for the typical PEO-bent double-chain-stranded tight-fit PPR. This particular pattern was similar to previously reported, single-chain-stranded loose-fit PPRs [10–12]. It suggested that with the further increase in the number of added γ-CDs, the resulting PEO-bent double-chain-stranded tight-fit PPRs tended to aggregate and settle out through the hydrogen bonding interaction. This leads to the breakdown of the core–shell micellar structure and the exposure of uncoated PMA chains to water. Under this circumstance, the entrapped γ-CDs began to slip off and return to accommodate the PMA segments, moving over them in a randomly distributed manner along the pentablock copolymer chain. This generated the single-chain-stranded loose-fit PPRs showing no characteristic channel-like tight-fit structure. In comparison to PEP100M10CD and PEP100M30CD, a weak peak at 6.8° and other three main peaks at 2θ = 12.4°, 17.2° and 21.5° also appeared in the diffraction pattern of PEP100M15CD. It was shown that at a feed molar ratio 15:1 of γ-CD to PEP100M, the supramolecular structure of PEP100M15CD changed from the PEO-bent double-chain-stranded tight-fit PPR into the single-chain-stranded loose-fit one.

The WXRD patterns of the resulting PR-based multiblock copolymers are also shown in Figure 2. As can be seen, both PR30CD50P and PR30CD80P exhibited only a single broad diffraction peak, most likely due to those remainimg γ-CDs randomly distributed along the pentablock copolymer chain to form an irregular noncrystalline state. This pattern was also observed in our recent reports [13–15].

#### ^1^H NMR and GPC analyses

**Figure 3:**
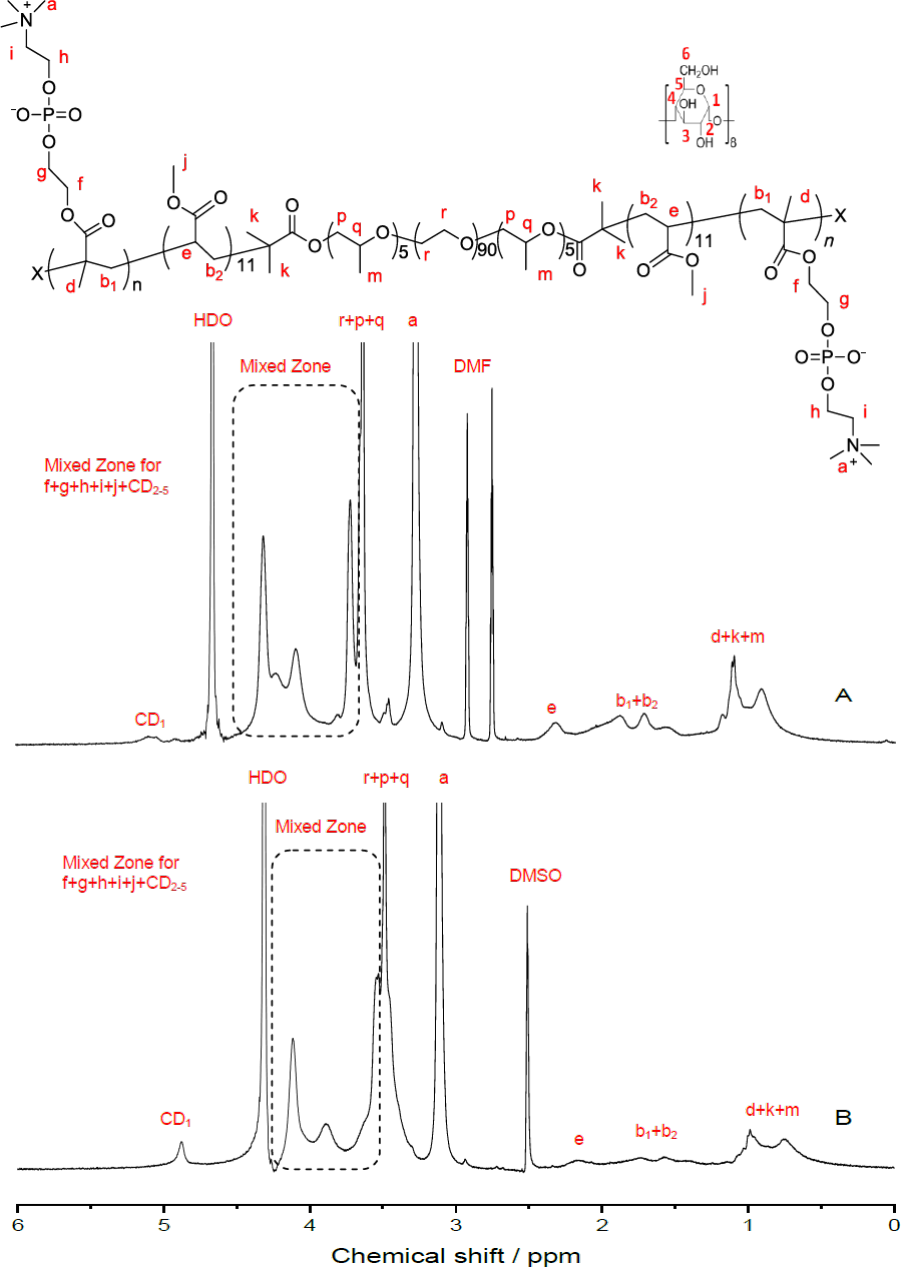
^1^H NMR spectra of PR30CD80P in DMF-*d_7_*/D_2_O (A) and DMSO-*d*_6_/D_2_O (B).

As the PMPC segments in the PRs were insoluble not only in DMF and DMSO but also in water, the ^1^H NMR spectra were measured in a mixed solution of DMF-*d*_7_/D_2_O (1/1, v/v) and DMSO/D_2_O (2/1, v/v), respectively, and the results are shown in Figure 3. The ^1^H NMR spectrum of the heptablock copolymer PR0CD30P is displayed in Figure S4, Supporting Information File 1. As can be seen, the proton resonance peak of γ-CD (CD_1_) is not easy to assign in DMF-*d*_7_/D_2_O as in DMSO/D_2_O. This suggests that the central PPR segment is soluble and the PMA segments are insoluble in this solvent. Accordingly, the DP of PMPC was determined from the integration area ratio of the proton resonance peaks of methyl groups (a) of MPC repeating units to that of the methylene protons in MPC and MA (b_1_ + b_2_) repeating units, according to Figure 3A. The CD coverage ratios were obtained from the integration area ratio of the proton resonance peaks of γ-CD (CD_1_) to that of methyl groups (a) of MPC repeating units from Figure 3B. It was found that only about one-fifth of the added γ-CDs were left on the PMA-PPO-PEO-PPO-PMA main chains after the second ATRP of MPC in this study. This was most likely due to a relatively smaller cross-sectional area of PMA compared with PHEMA, leading to a lower inclusion complexation stability of PMA-PPO-PEO-PPO-PMA with γ-CDs during the one-pot in situ ATRP of MPC [14].

**Figure 4:**
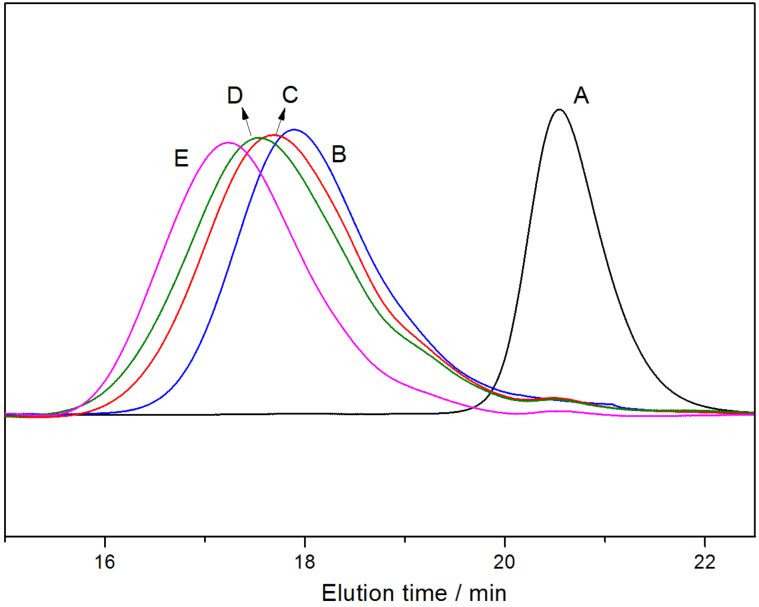
GPC curves of γ-CD (A), PR0CD30P (B), PR30CD30P (C), PR30CD50P (D) and PR30CD80P (E).

GPC analysis further confirmed the unique structure of the resultant γ-CD-based PRs. As depicted in Figure 4, the molecular weights were increased with the feed molar ratio of MPC to PEP40M, and all samples exhibited a symmetrical and unimodal GPC curve showing no free γ-CD peak. Although PMA was used as the outer hydrophobic segment to induce the self-aggregation of the pentablock copolymers in aqueous solution, a multimodal molecular weight distribution (which usually results from the addition of methacrylates to the polymers of acrylates) was not shown in the GPC curves of the PR-based multiblock copolymers [18–19]. Given that nearly the same quantity of 5–8 γ-CDs were left after the second ATRP (as determined by ^1^H NMR), it is suggested that the one-pot endcapping via the in situ ATRP of MPC using Cu(I)Br/Me_6_TREN as a catalyst successfully converted the γ-CD/PMA-PPO-PEO-PPO-PMA PPRs into the same conformational, mechanically interlocked, PR-based multiblock copolymers.

#### ^13^C CP/MAS NMR and 2D NOESY NMR testing

**Figure 5:**
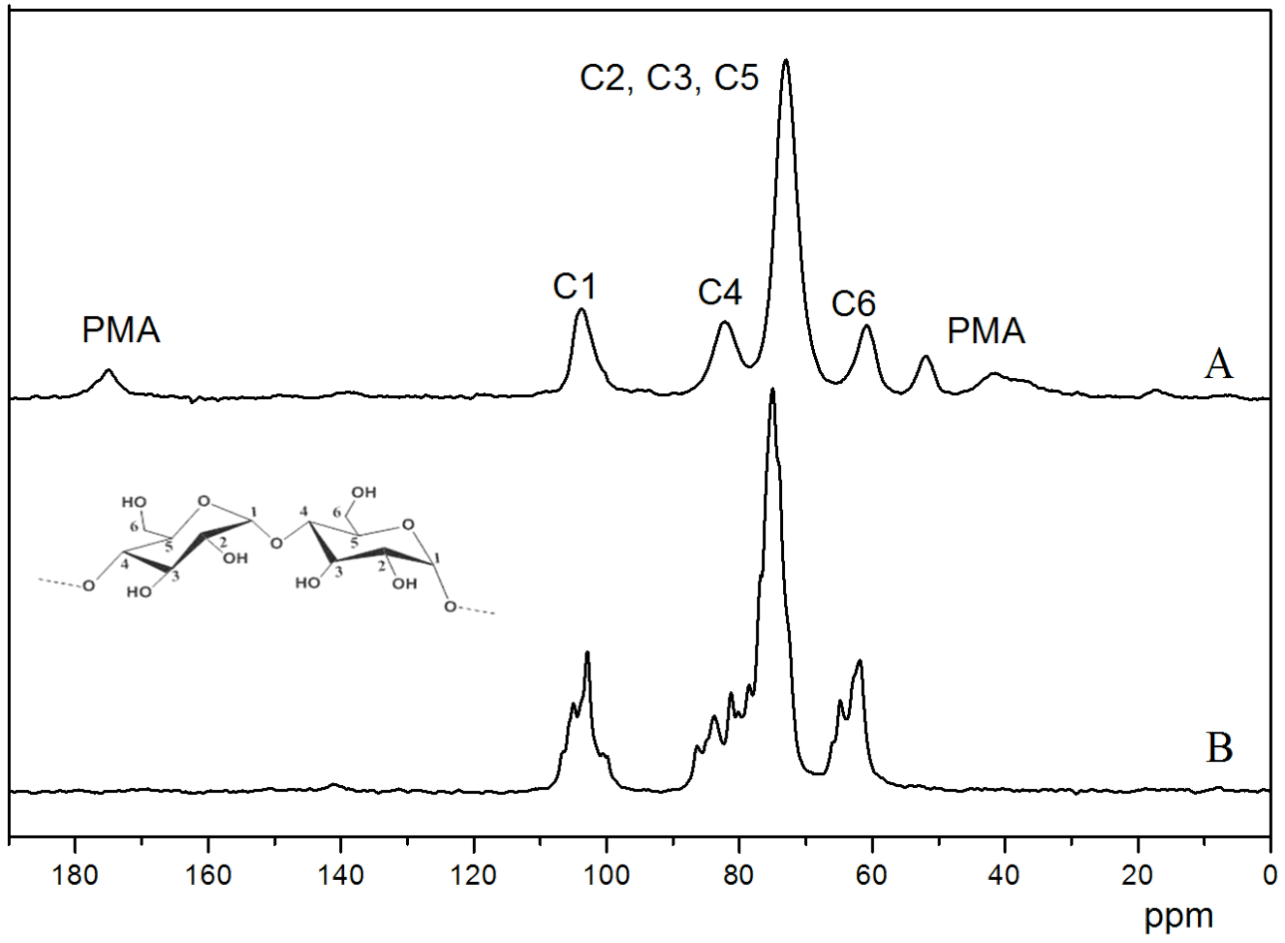
^13^C CP/MAS NMR spectra of PEP100M15CD (A) and γ-CD (B).

Figure 5 shows the solid-state ^13^C CP/MAS NMR spectra of the PEP100M15CD PPR sample and γ-CDs. In line with previous research [20], the less symmetric, cyclic conformations of γ-CDs in the uncomplexed crystalline state bring about its spectrum with multiple, clear C1, C4 and C6 resonance peaks. In the test sample, PEP100M15CD, the corresponding carbon reveals a single resonance peak together with the typical resonance peaks from PMA. This clearly suggested that the γ-CDs were threaded in a head-to-head and tail-to-tail fashion onto the PMA-PPO-PEO-PPO-PMA chain. This offered direct evidence, confirming the self-assembly of γ-CDs with PMA-PPO-PEO-PPO-PMA to give rise to PPRs.

**Figure 6:**
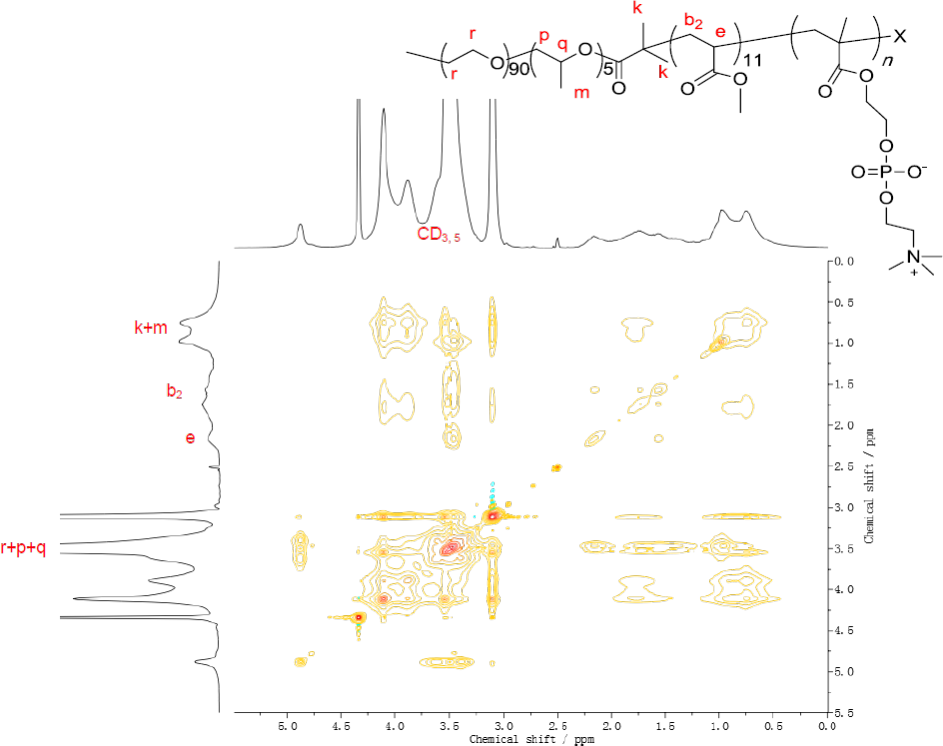
2D NOESY NMR spectrum of PR30CD80P in DMSO-*d*_6_/D_2_O.

To further confirm the inclusion complexation structure of γ-CDs with the pentablock copolymers and the preferential location of γ-CDs on the different segments after the second in situ ATRP, 2D ^1^H NOESY NMR measurements were carried out on the PR sample PR30CD80P (Figure 6). Consistent with ^1^H NMR and GPC analyses, the correlation of peaks between the interior protons of γ-CDs (CD_3_ and CD_5_) and those of MA (b_2_), PPO (m, p, q) and PEO (r) clearly indicated that host γ-CDs remained for inclusion of the guest pentablock copolymer chain after the one-pot ATRP of MPC. Because this spectrum was taken in DMSO-*d*_6_/D_2_O, the entrapped γ-CDs showed no bias to be located along a particular segment of the PMA-PPO-PEO-PPO-PMA chain.

#### FTIR measurements

**Figure 7:**
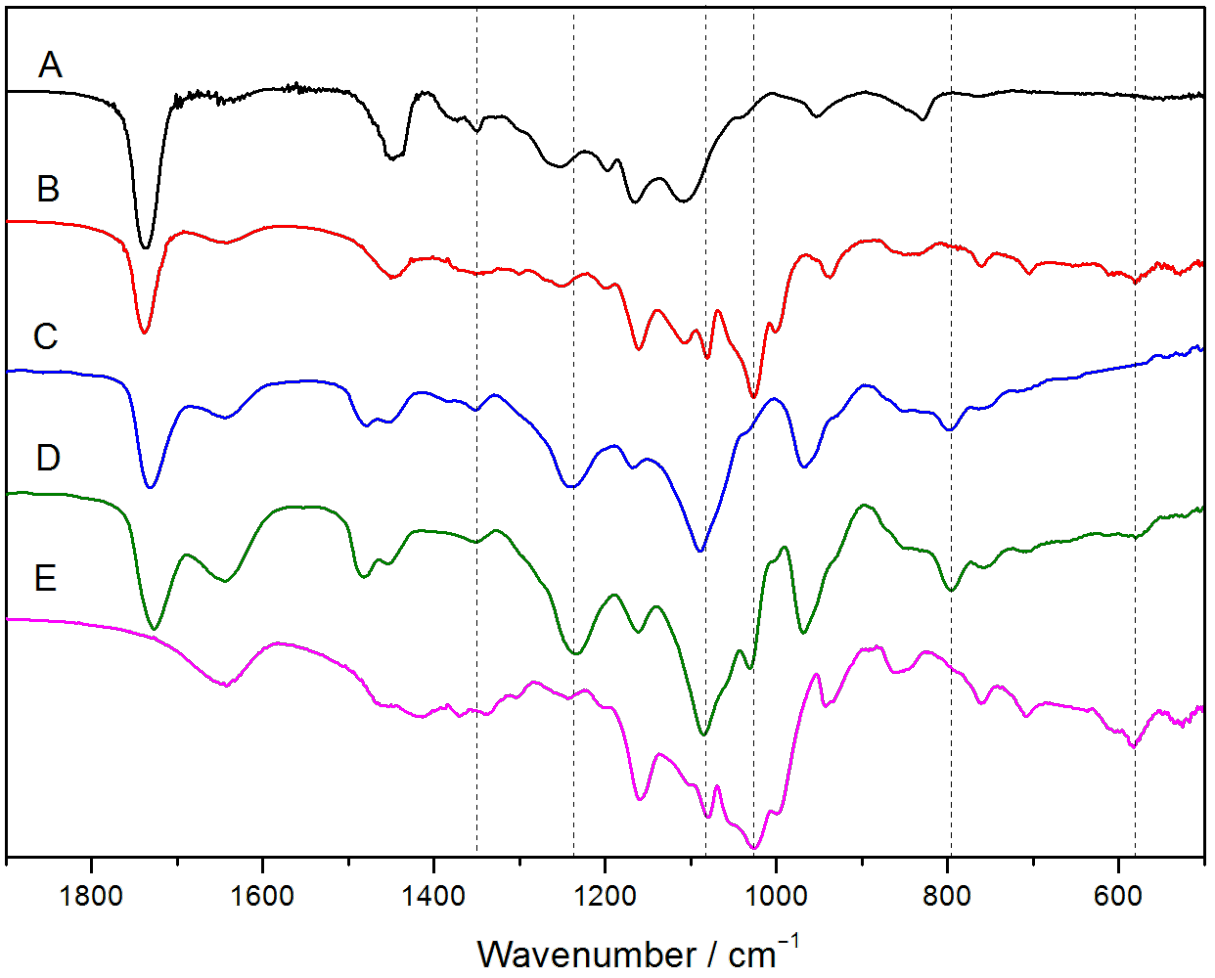
FTIR spectra of PEP100M (A), PEP100M15CD (B), PR0CD30P (C), PR30CD80P (D) and γ-CD (E).

The FTIR spectra of the PPR and PR samples and their precursors are depicted in Figure 7. The peaks at 1235, 1088 and 788 cm^−1^ are attributed to the stretching vibrations of O–P–O, C–N–C and P–O–C in MPC repeat units, respectively [21]. The wider absorption peak at 584 cm^−1^ (as compared with the uncomplexed γ-CD) and the characteristic peak at 1028 cm^−1^ were visible in the spectra of both PEP100M15CD and PR30CD80P, while the –CH_2_– vibration absorption peak of PEO at 1350 cm^−1^ disappears in the spectrum of PEP100M15CD. This is due to the restricting and shielding effects from the inner cavity of γ-CDs against the vibration of the corresponding chemical bond [22]. This confirmed that the PMPC segments were successfully attached to two ends of the pentablock polymer to convert γ-CD/PMA-PPO-PEO-PPO-PMA PPRs into the PR-based multiblock copolymers [14,23].

#### Thermal analysis

**Figure 8:**
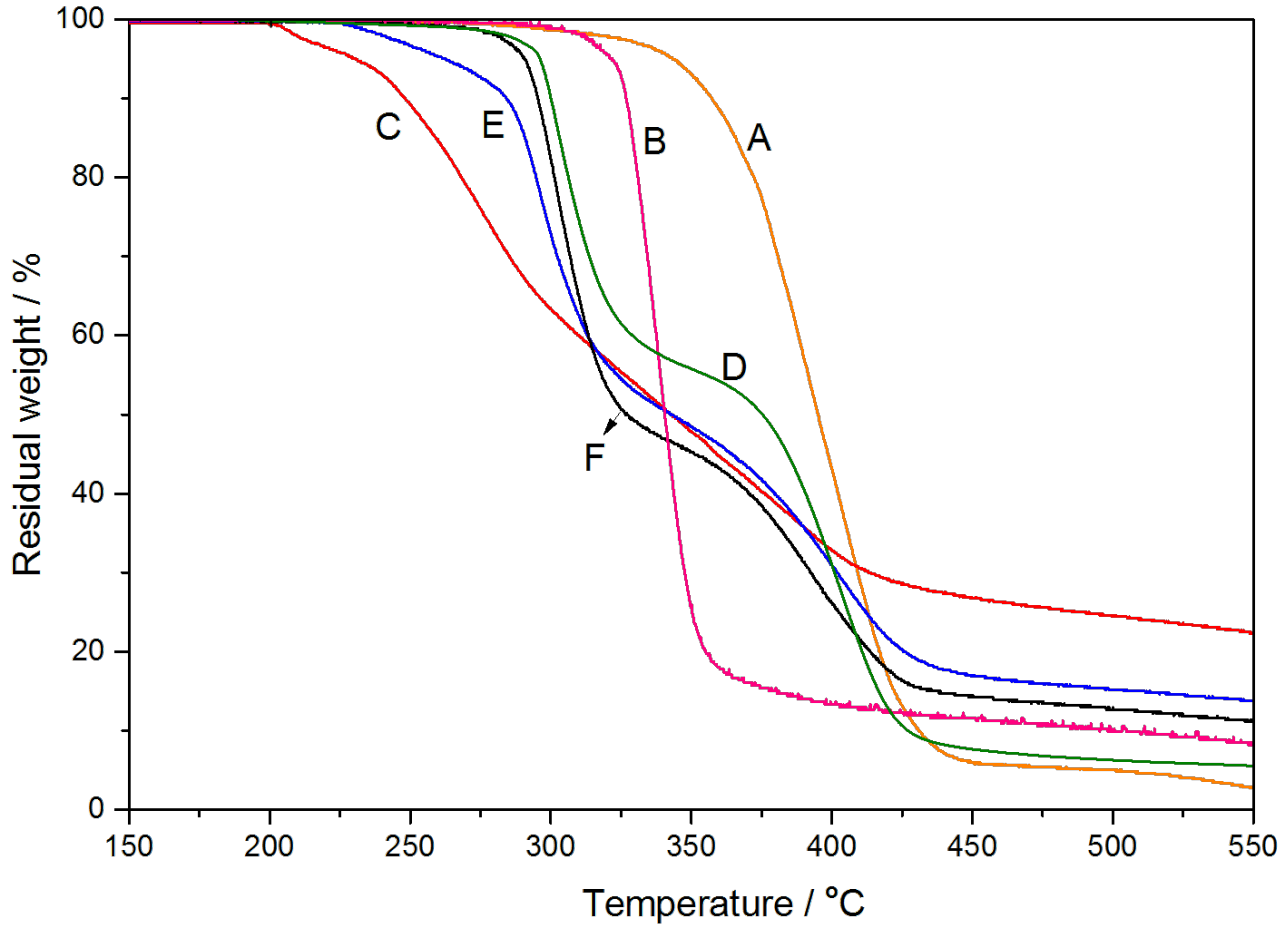
TGA curves of PEP100M (A), γ-CD (B), PEP15CD (C), PEP100M10CD (D), PEP100M15CD (E) and PEP100M30CD (F).

TGA analysis was carried out to further confirm the structure of the PPRs. As shown in Figure 8, the free γ-CD began to decompose at about 300 °C and the PEP100M at around 325 °C. Unlike either the pure γ-CD or PEP100M, the PEP100M10CD and PEP100M30CD samples underwent a two-step, thermal degradation process. The thermal weight loss at about 275 °C and 350 °C is attributed to the decomposition of γ-CD and the pentablock polymer chain, respectively. The pentablock copolymers were substantially stabilized by the formation of PPRs. Meanwhile, PEP15CD presented an additional thermal weight loss component starting at about 200 °C, possibly due to the decomposition of 2-bromoisobutyryl ends of the BrPEPBr in a U-shape [13]. Compared with the thermal decomposition of γ-CD, the PPR samples (including PEP15CD) displayed shifted, γ-CD-related thermal decomposition towards lower temperature. This was likely due to the differing architecture of the PPRs self-assembled from PMA-PPO-PEO-PPO-PMA with the varying amount of γ-CDs [10–12].

**Figure 9:**
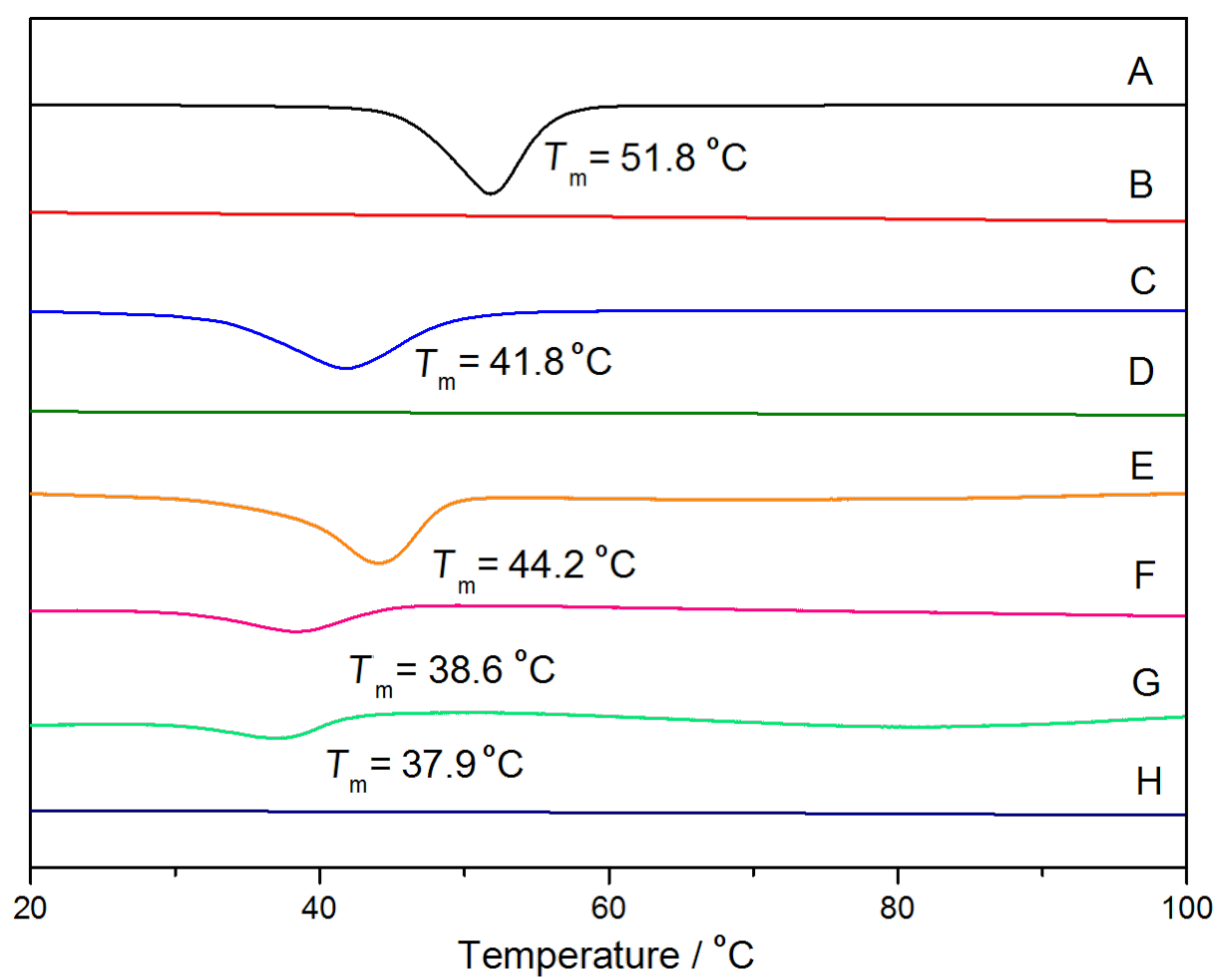
BrPEPBr (A), PEP15CD (B), PEP100M (C), PEP100M15CD (D), PR0CD30P (E), PR30CD50P (F), PR30CD80P (G) and γ-CD (H).

The DSC measurements of PPRs and PRs were also conducted in this study. As shown in Figure 9, BrPEPBr reveals an endothermic peak at 51.8 °C, corresponding to the melting point of the PEO crystalline phase. As for PEP100M, a lower melting point appeared at 41.8 °C, due to the interference of the PMA blocks attaching to the two ends of PPO-PEO-PPO. However, compared with the macroinitiator, the penta-block copolymer and pure γ-CD, the PEP100M15CD and other PPR samples exhibited no endothermic peak in the range from 20 to 100 °C. This clearly indicated that the entrapped γ-CDs restrict the central PEO segment from aggregating to form the crystalline phase [24]. The neat pentablock copolymer PR0CD30P gave rise to an endothermic peak for PEO at 44.2 °C. At the same time, weak endothermic peaks at 38.6°C and 37.9°C were observed in two PR samples, PR30CD50P and PR30CD80P, respectively. Given that nearly the same amount of γ-CDs were left on the polymer chain after the second in situ ATRP, the DP of PMPC segments seemed to exert little effect on the crystalline behavior of the central PEO segment in the PR-based multiblock copolymers. The occurrence of broad, weak endothermic peaks in PR30CD50P and PR30CD80P suggested that the remaining γ-CDs were randomly distributed along the pentablock copolymer chain after endcapping via the one-pot ATRP of MPC.

## Conclusion

Novel PPRs were synthesized in high yield from the self-assembly of γ-CDs with PMA-PPO-PEO-PPO-PMAs. It was found that at a lower γ-CD feed ratio, the central PEO segment was preferentially bent to pass through the cavity of γ-CDs to construct double-chain-stranded tight-fit PPRs. By further increasing the feed ratio, the added γ-CDs began to include the PMA segments and move over them in a randomly distributed manner along the pentablock copolymer chain to give rise to single-chain-stranded loose-fit PPRs. Moreover, these single-chain-stranded loose-fit PPRs were endcapped via the second in situ ATRP of MPC into the same conformational PR-based multiblock copolymers. The PPR and PR supramolecular polymers show potential as dynamic-responsive materials, carriers for controlled drug release, biosensors and catalysts.

## Experimental

### Materials

PPO-PEO-PPO with a central block of 90 PEO units and two flank blocks of 5 PPO units was supplied by Zhejiang Huangma Chemical Industry Group Co., Ltd., China. The average molecular weight (*M*_n_) is 4580 g/mol. Methyl acrylate (MA) was purchased from J&K Company, China, and was used after removal of inhibitors. Both 2-bromoisobutyryl bromide (BIBB) and *N*,*N*,*N*’,*N*”,*N*”-pentamethyldiethylenetriamine (PMDETA) were available from Sigma-Aldrich, USA. γ-Cyclodextrin (γ-CD) and 4-dimethylaminopyridine (DMAP) were supplied by TCI, Japan. Triethylamine (TEA) was purchased from VAS Chemical Reagents Company, China and refluxed with *p*-toluenesulfonyl chloride and distilled. 2-Methacryloyloxyethyl phosphorylcholine (MPC) was supplied by Joy Nature, China and used as received. Tris(2-(dimethylamino)ethyl)amine (Me_6_TREN) was obtained from Alfa Aesar, USA. Copper(I) chloride (Cu(I)Cl) and copper(I) bromide (Cu(I)Br) were purified by stirring in hydrochloric acid and acetic acid, respectively, and washed with deionized water, methanol, and ether, then finally dried and then stored under a nitrogen atmosphere. CH_2_Cl_2_ was stirred with CaH_2_ and distilled before use. All other solvents and reagents were of analytical grade.

### Synthesis of macroinitiator (BrPEPBr)

The macroinitiator was prepared as follows. PPO-PEO-PPO (2 mmol, 9.16 g) was dissolved in 20 mL CH_2_Cl_2_ in a 100 mL round-bottom three-necked flask. Thereafter, DMAP (4 mmol, 488 mg) and TEA (4 mmol, 404 mg) dissolved in 10 mL CH_2_Cl_2_ was added. 15 mL of CH_2_Cl_2_ containing 1.84 g 2-bromoisobutyryl bromide (8 mmol) was then slowly added to the mixture over 2 h at 0 °C under nitrogen atmosphere. Thereafter, the reaction continued for another 24 h at room temperature. The mixture was dissolved in THF and filtered three times to remove the ammonium salt. Finally, the crude product was precipitated in 500 mL anhydrous ether at 5 °C and then dried under vacuum to give a yield of 81.5%.

### Synthesis of PMA-PPO-PEO-PPO-PMA via ATRP

A strategy for the preparation of pentablock copolymer PMA-PPO-PEO-PPO-PMA with a feed molar ratio of BrPEPBr/MA equal to 1:100 was as follows. BrPEPBr (0.2 mmol, 0.976 g) was dissolved in 8 mL of DMF in a sealable Pyrex reactor. Subsequently, 1.72 g MA (20 mmol) and 0.138 g PMDETA (0.8 mmol) were added to the mixture and then quenched in liquid nitrogen. Before 79.2 mg CuCl (0.8 mmol) was added, the system was degassed with three freeze–vacuum–thaw cycles and purged with nitrogen. Then the reactor was sealed under vacuum and the polymerization started under stirring for 48 h at room temperature. Afterwards, the whole content was dissolved in THF and passed over a basic alumina column to remove the Cu salts. Finally, the crude product was precipitated in anhydrous ether and dried under vacuum.

### Preparation of BrPEPBr-γ-CD PPR

The saturated aqueous solution of γ-CD (486 mg, 0.375 mmol) was added to 2 mL of an aqueous solution of BrPEPBr (0.025 mmol, 122 mg,) under vigorous stirring, followed by stirring at 25 °C for 1 h at room temperature. A white PPR slurry was formed as a result of the self-assembly of γ-CDs with the macroinitiator. After washing with a small amount of water, white powder products were obtained by freeze-drying after centrifugation.

### Preparation of PMA-PPO-PEO-PPO-PMA-γ-CD PPRs

A protocol for the synthesis of PPRs via the self-assembly of PMA-PPO-PEO-PPO-PMA with γ-CDs (maintaining a feed molar ratio of PEP100M/γ-CD at 1:10) was as follows. The saturated aqueous solution of 0.26 g γ-CDs (0.2 mmol) was added to a 1 mL aqueous solution of 0.19 g PEP100M (0.02 mmol). The mixture was stirred for 1 h at room temperature, followed by storage at 6–8 °C in a refrigerator for 24 h. A white gel was formed as a result of the self-assembly of γ-CDs and pentablock copolymer. The gel was washed three times with distilled water and freeze dried to get the PPRs powder products.

### One-pot preparation of PRs

A protocol for the one-pot synthesis of PRs by endcapping PPRs via the second in situ ATRP of MPC was as follows. In a sealable Pyrex reactor, the mixture of 0.135 g of PEP40M (0.02 mmol) and 0.778 g of γ-CDs (0.6 mmol) in 3 mL water was stirred for 24 h at room temperature. The predetermined amount of MPC and 0.0184 g of Me_6_TREN (0.08 mmol) were added to the resulting suspension of PPRs and then quenched in liquid nitrogen. After the system was degassed with seven freeze–vacuum–thaw cycles and purged with nitrogen, 11.5 mg of CuBr (0.8 mmol) was quickly added. The reactor was sealed under vacuum and the reaction was maintained for 60 h at room temperature. After breaking the reactor, the product was dissolved in DMSO/H_2_O (1:1 v/v) and dialyzed against distilled water using a cellulose membrane (MWCO 3500) for 10 days, changing the water every 6 h, and then freeze dried.

### Measurements

^1^H NMR spectra were obtained from a Bruker ARX 400 MHz spectrometer at room temperature with tetramethylsilane (TMS) as the internal standard. The GPC analysis was conducted with a HLC-8320GPC (TOSOH, Japan) instrument at 30 °C at a flow rate of 0.3 mL/min. The solid-state ^13^C CP/MAS NMR measurements were carried out at 75 MHz with a spinning rate of 5 kHz at room temperature using a Bruker AV-300 NMR spectrometer. The chemical shifts were referred to an external adamantane standard. Wide angle X-ray diffraction (WXRD) patterns were recorded on a Shimadzu XD-D1 X-ray diffractometer with Ni-filtered Cu Kα (1.54 Å) radiation (20 kV, 40 mA). Powder samples were scanned from 2θ = 4.5–60° at a speed of 5°/min. FTIR spectra were measured using a Shimadzu IR Prestige-21 FTIR spectrometer at room temperature using the KBr pellet method. TGA analysis was performed with using a TA SDT 2960 instrument at a heating rate of 10 °C/min from room temperature to 500 °C in a nitrogen atmosphere. DSC measurements were measured on a SHIMADZU DSC-60 differential scanning calorimeter with a scanning temperature range from 20–80 °C at a scanning rate of 10 °C/min. The transmission electron microscopy image was observed using a JEM 1200EX (JEOL) transmission electron microscope operating at 120 KV.

## Supporting Information

The Supporting Information File 1 contains ^1^H NMR spectra of BrPEPBr and PEP100M, GPC curves of BrPEPBr, PEP40M, PEP60M and PEP100M, TEM image of PMA-PPO-PEO-PPO-PMA in water and ^1^H NMR spectra of PR0CD30P.

File 1 Additional experimental data.
