# Supplementary material for: Self-assemblies of γ-CDs with pentablock copolymers PMA-PPO-PEO-PPO-PMA and endcapping via atom transfer radical polymerization of 2-methacryloyloxyethyl phosphorylcholine
Source: Beilstein J Org Chem. 2015 Nov 23;11:2267–77. doi: 10.3762/bjoc.11.247 (PMC4685896; doi:10.3762/bjoc.11.247)
Supplement: File 1 — Additional experimental data. [file Beilstein_J_Org_Chem-11-2267-s001.pdf]

Supporting Information

for

Self-assemblies of γ-CDs with pentablock copolymers PMA-PPO-PEO-PPO-PMA and end-capping via atom transfer radical polymerization of 2-methacryloyloxyethyl phosphorylcholine

Jing Lin, Tao Kong, Lin Ye, Ai-ying Zhang and Zeng-guo Feng*

Address: School of Materials Science and Engineering, Beijing Institute of Technology, Beijing 100081, China

Email: Zeng-guo Feng - sainfeng@bit.edu.cn

*Corresponding author

Additional experimental data

Contents

^{1}H NMR spectra of BrPEPBr and PEP100M.

GPC curves of BrPEPBr, PEP40M, PEP60M and PEP100M.

TEM image of PMA-PPO-PEO-PPO-PMA in water.

^{1}H NMR spectrum of PR0CD30P.

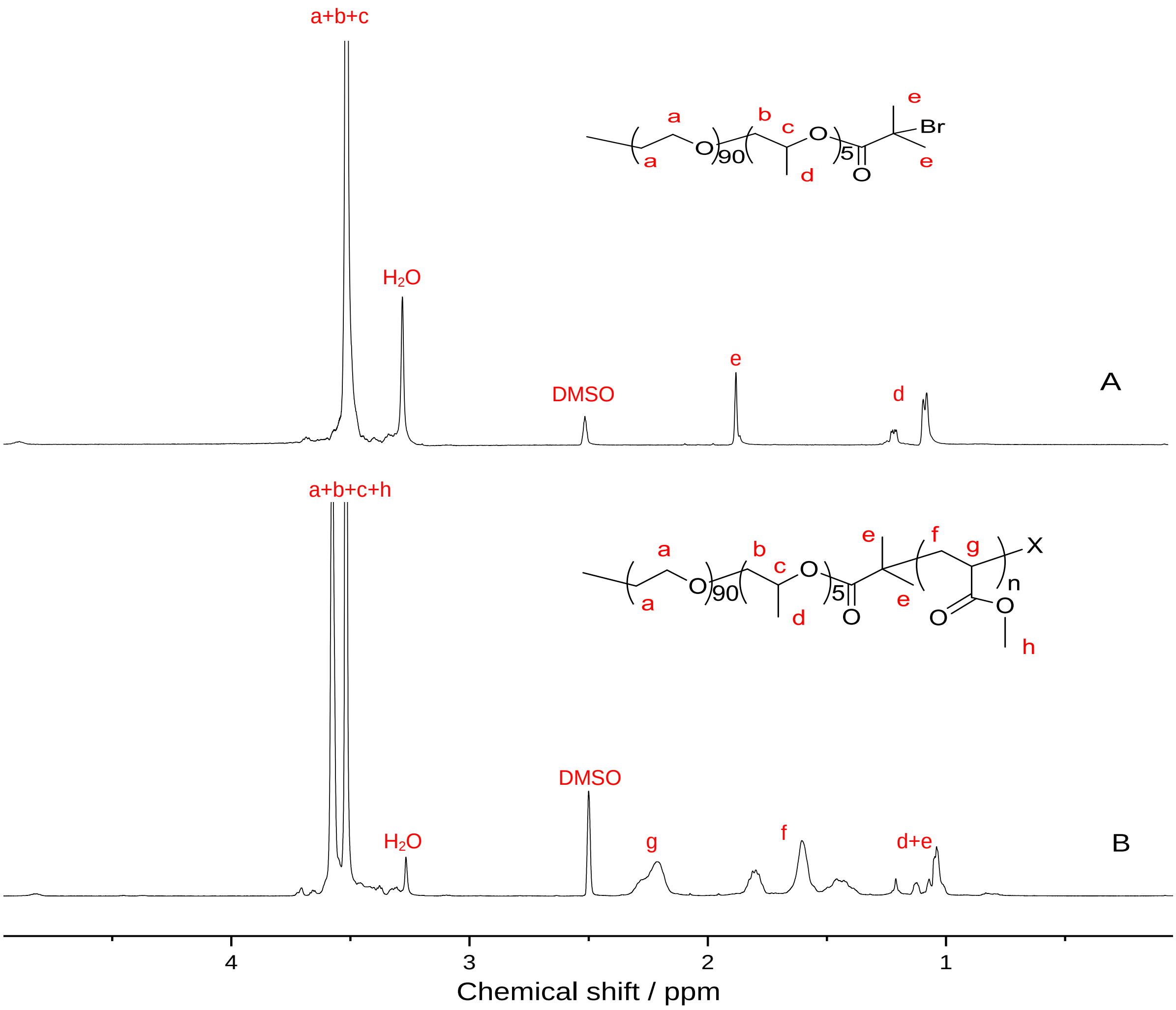

Figure S1: ^{1}H NMR spectra of BrPEPBr (A) and PEP100M (B).

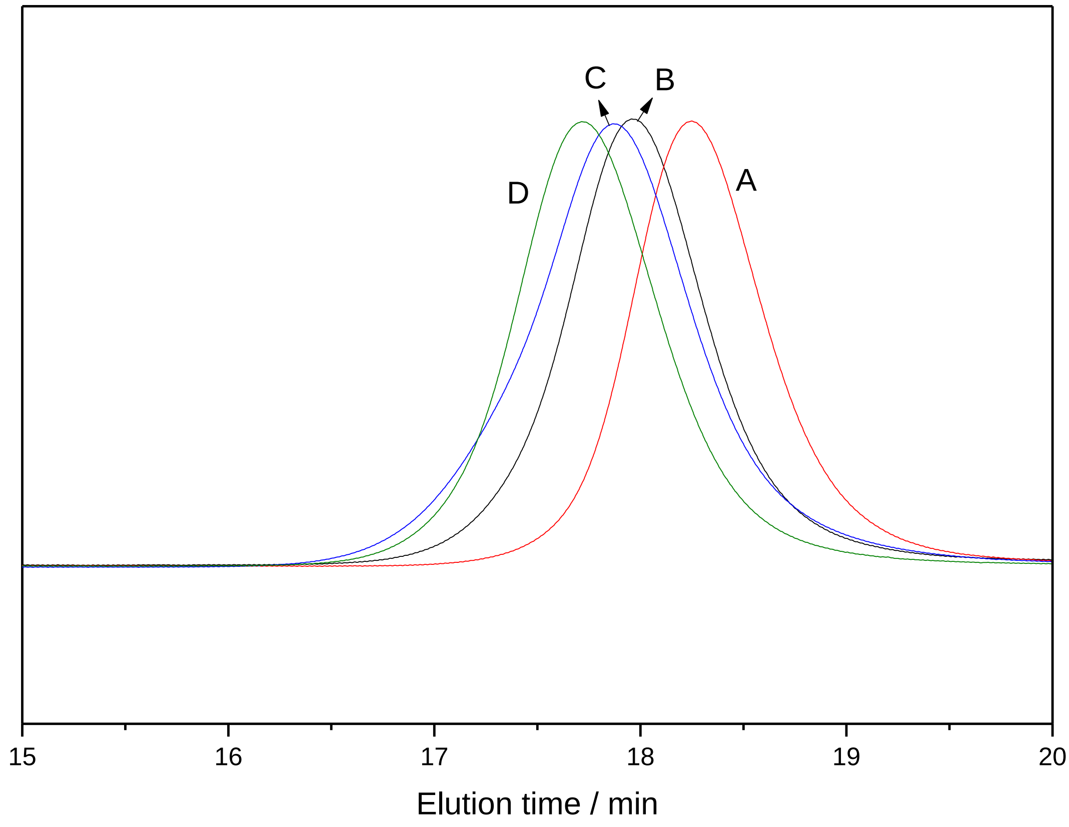

Figure S2: GPC curves of BrPEPBr (A), PEP40M (B), PEP60M (C) and PEP100M (D).

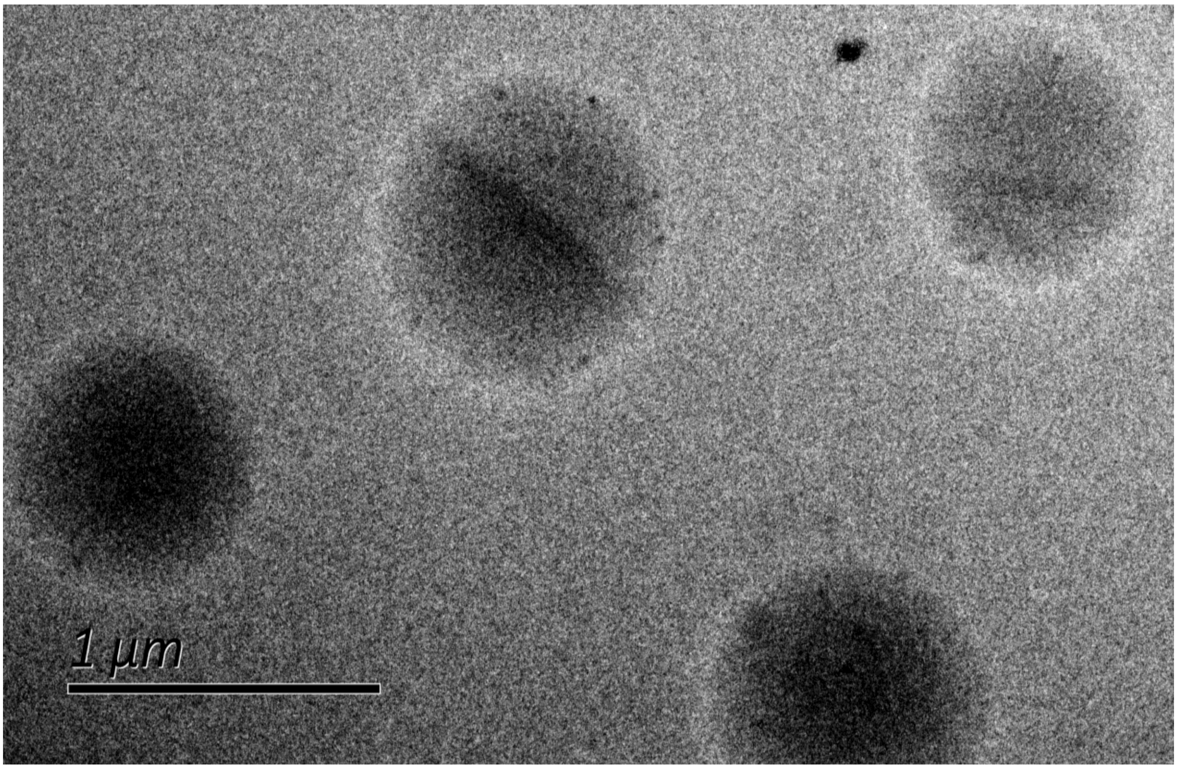

Figure S3: TEM image of PMA-PPO-PEO-PPO-PMA in water.

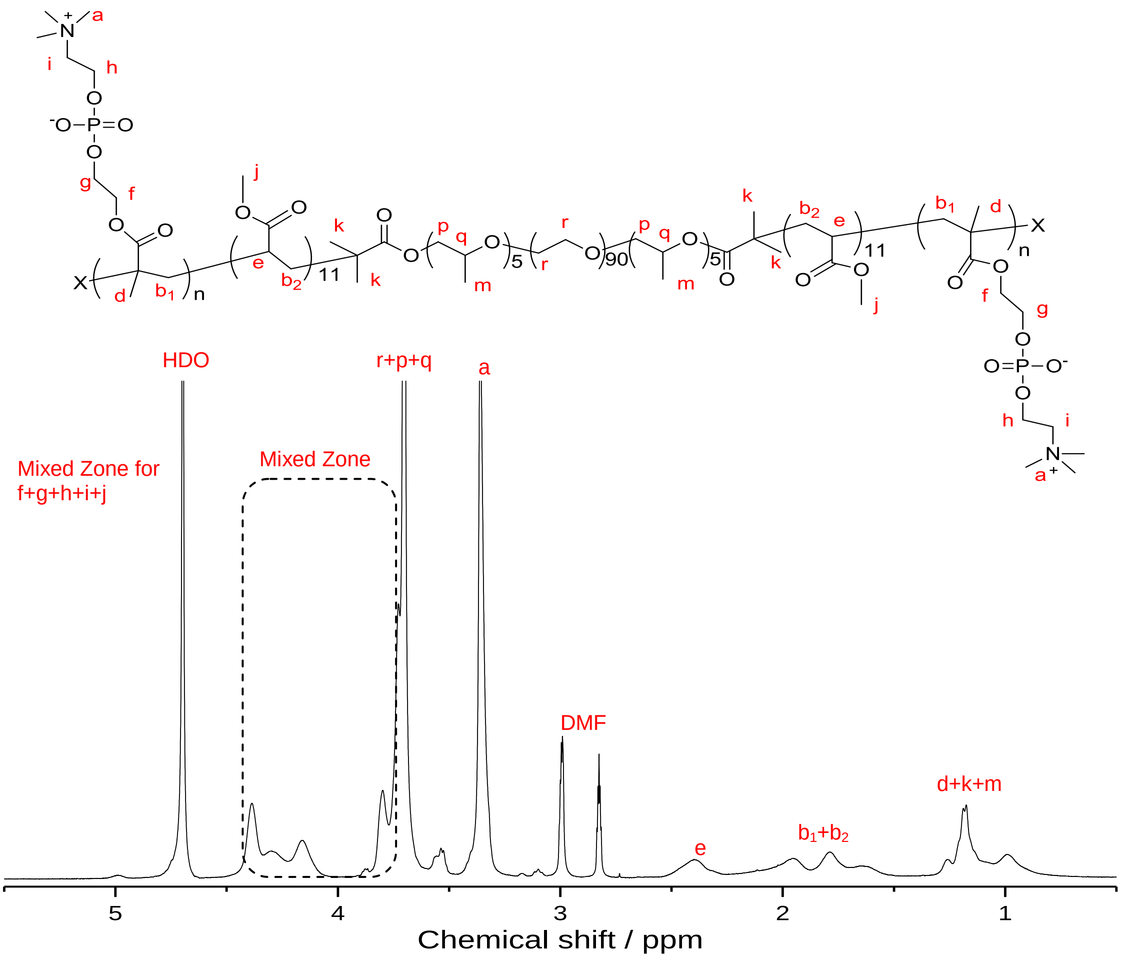

Figure S4: ^{1}H NMR spectrum of PR0CD30P.
